# Cornerball: a new alternative sport proposal for school physical education

**DOI:** 10.3389/fspor.2024.1360123

**Published:** 2024-05-13

**Authors:** Pelayo Diez-Fernández, Brais Ruibal-Lista, David Revesado-Carballares, Alberto Rodríguez-Cayetano, Sergio López-García

**Affiliations:** ^1^ Facultad de Educación, Universidad Pontificia de Salamanca, Salamanca, España; ^2^ Grupo de Investigación en Actividad Física, Deporte y Salud (GIADES), Facultad de Educación, Universidad Pontificia de Salamanca, Salamanca, Spain; ^3^ EUM Fray Luis de León, Universidad Católica de Ávila, Valladolid, España; ^4^ Facultad de Educación, Universidad de Salamanca, Salamanca, España

**Keywords:** alternative sports, cornerball, physical education, participation, game

## Abstract

Physical Education has had to evolve and change throughout history to adapt to the demands of society. As a result, teachers have had to seek pedagogical alternatives to ensure that students are active, motivated, and engaged in the classroom. This approach allows for the development of motor, cognitive, and socio-emotional skills in students, ultimately contributing to the holistic development of the individual, which is the primary goal of education. Out of this intrinsic need for Physical Education, Alternative Sports have emerged, providing opportunities for different types of learning compared to more traditional sports. Cornerball, a hybrid between split-court and wall-based sports, played in a somewhat unique playing area—a 90° corner, is introduced with the aim of offering a new Alternative Sport. The objective of this descriptive study is to present a new pedagogical proposal designed for the educational context, highlighting its characteristics and fundamental aspects to consider, such as rules, the playing field, participants, and methodological strategies for its implementation within a Physical Education classroom. Therefore, the aims and purposes of this work are to describe a new sports game so that in the future, more detailed and specific empirical studies can be proposed.

## 1 Introduction

The Physical Activity (PA) configures a mechanism of great importance for today's society. It contributes to raising awareness about the significance of movement in the well-being and health of the population. Through an active and healthy lifestyle, potential pathologies in the youngest can be prevented, obtaining benefits not only for physical health but also for psychological and behavioral aspects among students (1, 2). To achieve this, the educational system relies on a powerful tool: Physical Education (PE).

Over the past few years, PE has undergone modifications in its curricular elements (3) to become a more comprehensive model, adapting to modern times and addressing the educational needs posed in the 21st century (4). To adjust to these changes and requirements, PE teachers must seek new formulas to motivate their students, adapting basic content in their classes, such as motor skills or games and sports (5).

The primary purposes of the PE area within our country's educational system encompass physical-motor development, the creation and recreation of physical culture, and the comprehensive development of individuals (4); To achieve these aims, an element such as games is available. Games are understood as human activities that undergo modifications depending on the society or culture in which they develop. Through games, students expand their knowledge via physical-motor, cognitive, affective-emotional learning, teamwork, and value education (6–9). Similarly, play holds a very valuable cultural value from the perspective of those who engage in it, making it a powerful tool for Physical Education teachers to achieve holistic child development (10, 11). Given that sociomotor sports, containing a social component, fit perfectly into Physical Education classes, they foster valuable attitudes and social skills (10).

On the other hand, sport is considered one of the primary tools within PE to achieve its pedagogical objectives (12). Educational sport is one of the basic and most developed contents in PE (13). It should distance itself from high-performance sports by reducing competitiveness (14), focusing on transmitting values, and reinforcing regular practice of physical-sporting activity among students (15, 16).

However, certain sports considered “traditional,” characterized by excessive competition or regulation, fail to motivate students (17–19). In the same line, Gil-Madrona et al. (10) note that popular sports activities are very present in a child's motor biography, making it less likely to surprise and motivate them in PE classes. Therefore, alternative, inclusive, and challenging sports should be proposed to achieve maximum student participation, regardless of their abilities (20–22). From this necessity, arise sports known as “alternative sports” (AS). AS lacks a universally accepted definition due to a lack of standardization of their peculiar and individual characteristics encompassing all of them (23). AS share common elements such as active student participation, value transmission, flexible regulations, and the use of innovative, original, and alternative equipment (24–26), sidelining competitiveness and results to be more appealing to students (27).

Therefore, Feu (24) recommends the inclusion of this type of sport in PE classes. They describe it as a more educational approach since all students start from the same level of knowledge about the game and a more equal motor competence to generate new motor situations and, consequently, new learning.

Consequently, AS could be defined as those departing from the standard using physical-sporting practice, alternative materials, facilities, etc., as fundamental elements to achieve educational objectives and value transmission, enhancing student motivation, emphasizing cooperation over competition, thus promoting healthy lifestyle habits and an active lifestyle (13, 28, 29).

On the other hand, classifying these sports is somewhat complicated because, depending on their understanding, the type of sport, implements to use, time, participation, etc., various classifications exist, such as those by Ellis (30), Almond (31), or Méndez-Giménez (32), among others.

To locate this new proposal, Méndez-Giménez's (32) classification, a modification of Almond's proposal in (31), will be used. It proposes classifying sports into: invasion sports, wall or barrier sports, divided court sports, striking and fielding sports, moving target sports, and fixed target sports with or without opposition.

## 2 Alternative sports in physical education

The AS arise from the need to foster “sports for all,” diverging from the traditional archetype dictated by the requirements in high-performance sports and federative guidelines, wherein only those with better physical abilities fit (33). Consequently, AS prioritize cooperation, values, satisfying experiences, and, notably, the promotion and practice of PA (13). Furthermore, they modify rules or norms, as well as structural elements of traditional sports, to achieve comprehensive student development (27, 34).

Contemporary society demands the application of methodologies, adequate resources, training … in short, seeking alternatives to ensure that learning resonates with students and, thereby, achieve comprehensive individual development (35). Teachers design their sessions based on their experiences, training, available facilities… (36). Therefore, similar to other authors (25), it is proposed and defended to utilize AS in the educational context.

The application of different sports, termed “alternative,” yields a series of benefits supported and confirmed in scientific literature. Firstly, they provide physical benefits by increasing physical-sporting activity during the play of certain alternative sports compared to traditional ones (37–40). Additionally, concerning co-education and gender equality, through Quidditch (41, 42) or Korfball (43, 44), it has been demonstrated that social stereotypes decrease, providing more opportunities than other more traditional sports. Along the same lines, through Pinfuvote, it has been shown to contribute to the development of sporting attitudes and values, cooperation, teamwork, and, of course, healthy lifestyle habits (45–48).

Moreover, increased physical activity has been found in co-educational classes, with girls' participation standing out in playing Korfball (49) and Kinball (40). Related to this, students have preferred co-educational orientation in PE classes (50). Conversely, in other studies, it has been demonstrated that boys practice more in the sports field, regardless of the type of sport (51).

Furthermore, it has been evidenced that the use of an alternative sport vs. a traditional one favors student participation and motivation in PE classes (13, 52), additionally enhancing aspects related to involvement and motivation when applying teaching models like the Sports Education Model (SEM) (53).

Due to the surge of AS in the Physical Education classroom such as Ringol (20), Twincon (22), Goubak (21), Unihoc (29), Pickleball [Barranca-Martínez, Hernández-Beltrán, & Gamonales (54)], Brokenball (55), or Artzkirol (56) among others, the following is proposed in this document: to describe and detail the characteristics of a new alternative sport to be used in Physical Education classes and thereby achieve the acquisition of new learning and motor behaviors. Since, in each different game situation, one can appreciate the operation of different logics based on the structure of the game, its internal logic, or the possible relations of cooperation or opposition (57). Therefore, split-court sports have highlighted the constant interaction among players, thus promoting the development of cognitive and tactical skills. Likewise, wall and court sports challenge spatial perception and foster quick decision-making in the face of unpredictable rebounds. Hence, this proposal is highly suitable for generating motor learning by blending skills from both sports and utilizing key aspects of both types of sports.

However, in physical education, there are other sporting alternatives, ambivalent and with optimal playability, such as motor triads, which are those sports games that intermix 3 opposing teams, generating the phenomenon of the paradox of being able to cooperate with one team at a specific moment of the game or, on the contrary, oppose and collaborate with the opponent (58, 59). This alternative also holds high educational value by fostering communication among participants to achieve the teaching-learning process (60) and involving decision-making processes with a playful sense (61).

### 2.1 Pedagogical justification

As current legislation on Primary Education (62), states, “*one of the objectives of Physical Education at this educational stage is the approach to cultural manifestations of a motor nature and the development of all decision-making processes involved in the resolution of motor situations*” (62).

To achieve this, the execution of motor actions in different situations and spaces will allow the development of motor, cognitive, affective, and social skills.

To address this, the educational curriculum for Primary Education (ages 6–12) includes a content block entitled “*Problem-solving in motor situations*”, with a transdisciplinary approach aimed at developing individual, cooperative, oppositional, and collaboration-opposition motor actions (62), within which the proposal of this sport, Cornerball, is framed. We highlight:

Block C “Problem-solving in motor situations” (62).

• Decision making: Coordination of actions with peers in cooperative situations. Adjustment of action to the opponent's location in motor situations of pursuit and interaction with a mobile object.
• Perceptual-motor abilities in practice context: integration of body schema; body awareness; laterality and its projection in space; eye-foot and eye-hand coordination; static and dynamic balance.
• Conditional capacities: basic and resultant physical capacities (coordination, balance, and agility).

Furthermore, it presents another block, named “*Emotional self-regulation and social interaction in motor situations*”, which focuses on the development of social skills and the promotion of constructive and inclusive relationships among participants in these motor contexts (62). We highlight:

Block D “Emotional self-regulation and social interaction in motor situations” (62).

• Social skills: verbalization of emotions derived from interaction in motor contexts.
• Respect for game rules.
• Concept of sportsmanship.

Concept of fair play or “sportsmanship”. Similarly, legislation regarding Compulsory Secondary Education (12–16 years) also divides the Physical Education subject into the same content blocks (62).

Block C is also referred to as “Problem Solving in Motor Situations,” and Block D as “Emotional Self-regulation and Social Interaction in Motor Situations” (62).

Just like in Primary Education, the contents of these blocks are also suitable for development through our sport. We list the following:

Block C “Problem Solving in Motor Situations” (62):

• Decision-making: Group guidelines to optimize the group's motor resources for resolving the action/task in cooperative situations. Adapting one's own movements to the actions of the opponent in opposition situations.
• Delimitation of prior attack and defense strategies based on the characteristics of the team members in collaboration-opposition motor situations involving pursuit and interaction with a mobile object.
• Conditional capacities: development of basic physical capacities.
• Specific motor skills associated with technique in physical-sporting activities.

Block D “Emotional Self-regulation and Social Interaction in Motor Situations” (62):

• Emotional self-regulation: Mood control and failure management strategies in motor situations.
• Respect for rules: Fair play at different levels of sport and physical activity.
• Identification and rejection of behaviors contrary to coexistence in motor situations.

## 3 Cornerball: description of the proposal

Cornerball is presented as a hybrid recreational-sporting modality that blends two very characteristic types of sports: court-divided sports and wall sports. Both share common principles; they aim to send the mobile object to an opponent's area to gain an advantage or prevent its correct return (63).

Specifically, court-divided sports involve players positioned face-to-face, separated by a net (line, rope, or a “dead” zone), aiming to send the object over it to gain an advantage or hinder the opponent's return (64). Similarly, wall sports involve sending an object against a wall, exchanging hits indirectly (after contact with the wall), making it challenging for the opponent to return (64).

The shared principles between these two sports include alternative participation (no possession dispute over the object), opposition (one or more opponents with opposing intentions), spaces (separated in court-divided games or shared in wall sports where the wall is crucial for gameplay), game objectives (making the object touch the ground twice or having the opponent take it out of the field boundaries), the object itself (varying in sizes or materials), or the use of implements (utilizing one or both body segments) (64, 65).

Moreover, the sporting gesture of hitting (defensive and offensive) is a common technical element in both sports (64). In terms of shared tactical aspects, there is a need to send the object to a distant area of the field (66), move the opponent to a specific zone, leaving the field open, or exploit the opponent's weaknesses (64).

This document aims to showcase the development and implementation of a new alternative sport by modifying the main variables that make up the general structure of sports games (object, implements, net, spaces, time, players, and rules) (64).

### 3.1 Cornerball court

#### 3.2.1 Playing field

• The playing field consists of two walls forming a 90-degree angle.
• The playing area is delimited by an arc, drawn from wall to wall with an 8 m radius measured from the vertex formed by the two walls and the ground.
• The no-bounce zone after the serve is set at 2 m.
• The total area of the playing field is 50.26 square meters.
• The court is divided into 2 halves of 45 degrees by a net.
• The lines marking the arc (baseline), the no-bounce line, and the vertical line (wall) at the end of the field will be 5 cm wide and white.

#### 3.2.2 Net

**Figure 1:**
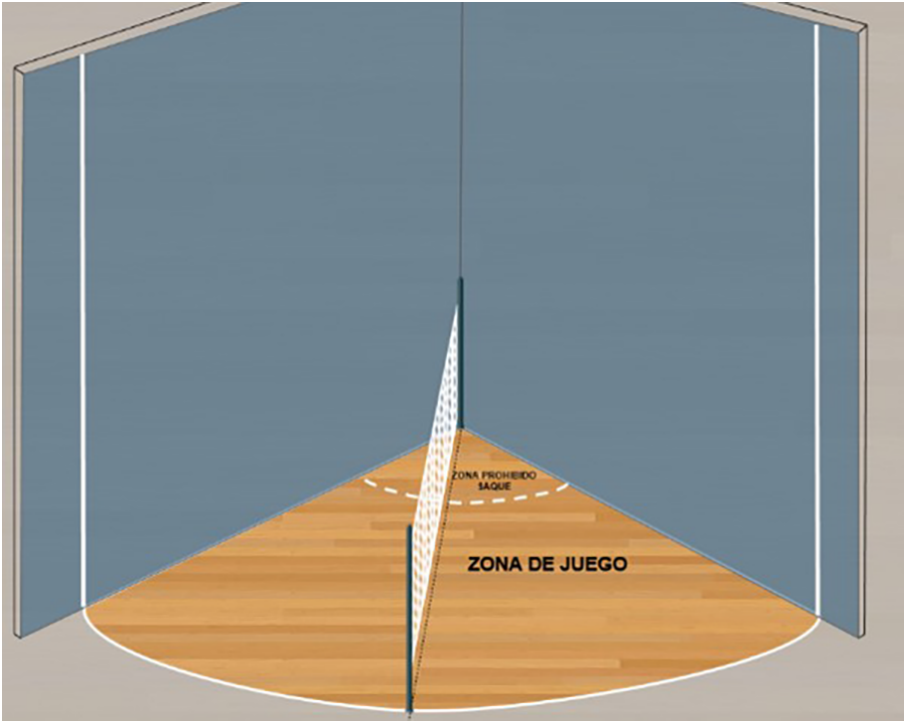
Playing field. Perspective 1.

• The net spans 8 m from the angle formed by both walls, dividing the field into 2 halves of 45 degrees.
• The net is suspended at 0.85 m above the ground, rising at its ends to a maximum of 0.90 m.
• The net is suspended using a metal cable with a maximum diameter of 0.01 m, connected at its ends to a pole that can have a maximum height of 0.85–0.95 m and can be anchored to the ground for national or international competitions. Alternatively, at the vertex between the two vertical walls, the net can be connected to a hook at 0.85–0.90 m.
• For initial sports training or facilities not specifically prepared for Cornerball, the poles can be mobile and supported on the ground with horizontal supports.
• The net poles are positioned so that their outer surfaces align with the edges of the baseline and the corner. These poles may have a circular or square shape, but they must have rounded corners. Also, the pole placed in the corner must have a corner-shaped support foot to fit without obstructing the game (Figure 1).
• The net is finished with an upper band of 0.05 m width in white, and the cable securing the net runs through its interior.
• The net must be fully extended to occupy the entire space between the poles and the court surface, leaving no space between the net's ends and the poles.
• The threads will be made of artificial fibers, and the mesh size will be small enough to prevent the ball from passing through.

### 3.2 Cornerball materials

**Figure 2:**
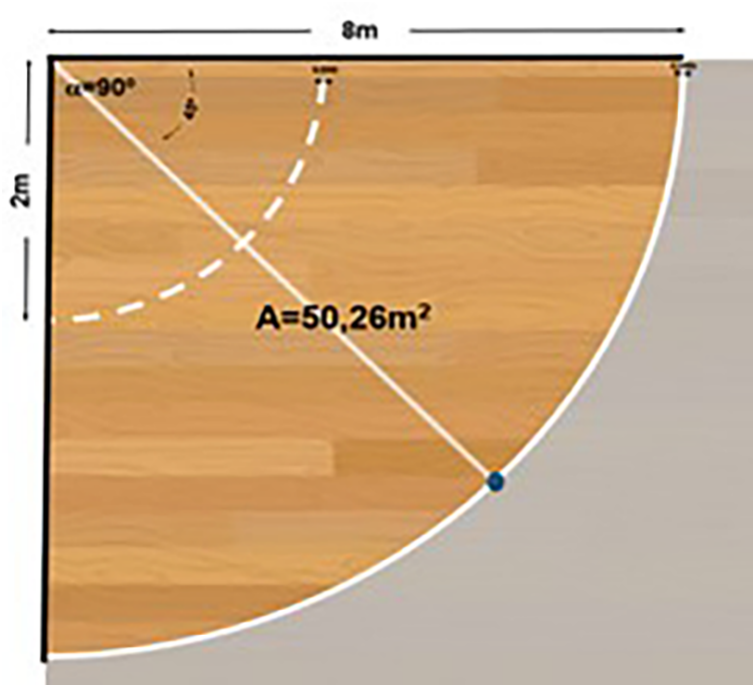
Playing field. Perspective 2.

**Figure 3:**
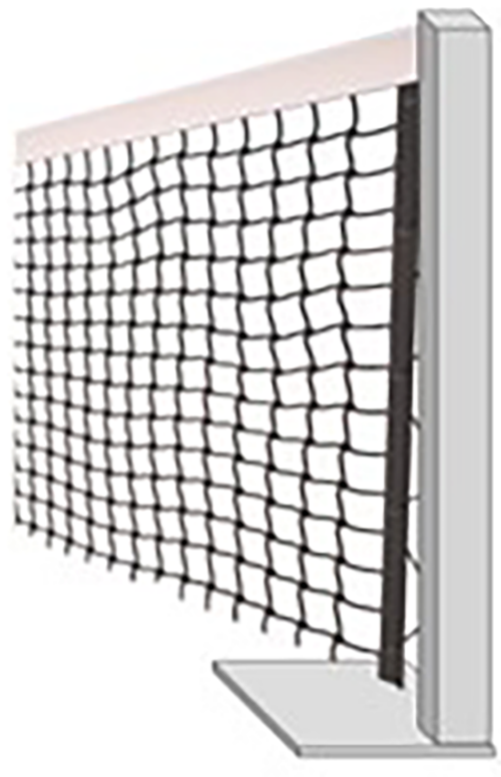
Cornerball net.

For the development of Cornerball, specific characteristics for the ball and paddle are necessary (Figures 2, 3).

The ball will be made of soft rubber, with a uniform surface in a striking color. Its dimensions should range between 5 cm and 6 cm in diameter (Ø), weighing between 45 and 50 g.

The paddle consists of two parts: the head and the handle in a single body. The paddle material should be rigid, non-compressible, such as plastic, layers of fiberglass, and/or carbon, providing stiffness and comfort. Additionally, the striking surface will be the same on both sides, which may be flat, smooth, or textured. It must have a non-elastic cord or strap for wrist attachment at the end of the handle as a safety precaution, which must be mandatory:

• Handle: The handle will have a length of 12 cm–15 cm, a width and thickness of 3 cm–5 cm.
• Head: The head's length, added to the handle's length, should not exceed 40 cm. It will have a maximum width of 18 cm–20 cm and a maximum thickness of 10 mm–15 mm.
• Weight: The weight will depend on the material used in construction.

### 3.3 Cornerball participants

Cornerball is an inclusive and innovative alternative sport in which athletes can compete individually or in pairs, although the preferred mode is mixed pairs.

Each pair of players will position themselves on each side of the net, located on opposite ends of the court. The player initiating the ball into play serves (server), and the one responding is the receiver (receiver).

The receiving player can position themselves anywhere in their court, as can their partner and the partner of the server, except during the serve when all 4 players must be outside the court.

Additionally, player participation must alternate; when a player hits and sends the object to the opposing court, they must immediately step back to allow their partner to take over the playing space. Furthermore, except during the serve, players can position themselves wherever they deem suitable for gameplay.

### 3.4 Cornerball regulation

#### 3.4.1 Rule 1: scoring system

The scoring system in Cornerball operates as follows: there is no time limit, and the match consists of 3 sets of 15 points, without a points advantage. The first team to win two sets wins the match. The unique aspect of the scoring system in this new alternative sport is that both teams start at 15 points, and the first to reach zero wins.

#### 3.4.2 Rule 2: the serve

The court sides will be determined by a draw before the match begins, and sides will change after each set. The first serve of each set always starts from the left side of the court, facing the corner.

**Figure 4:**
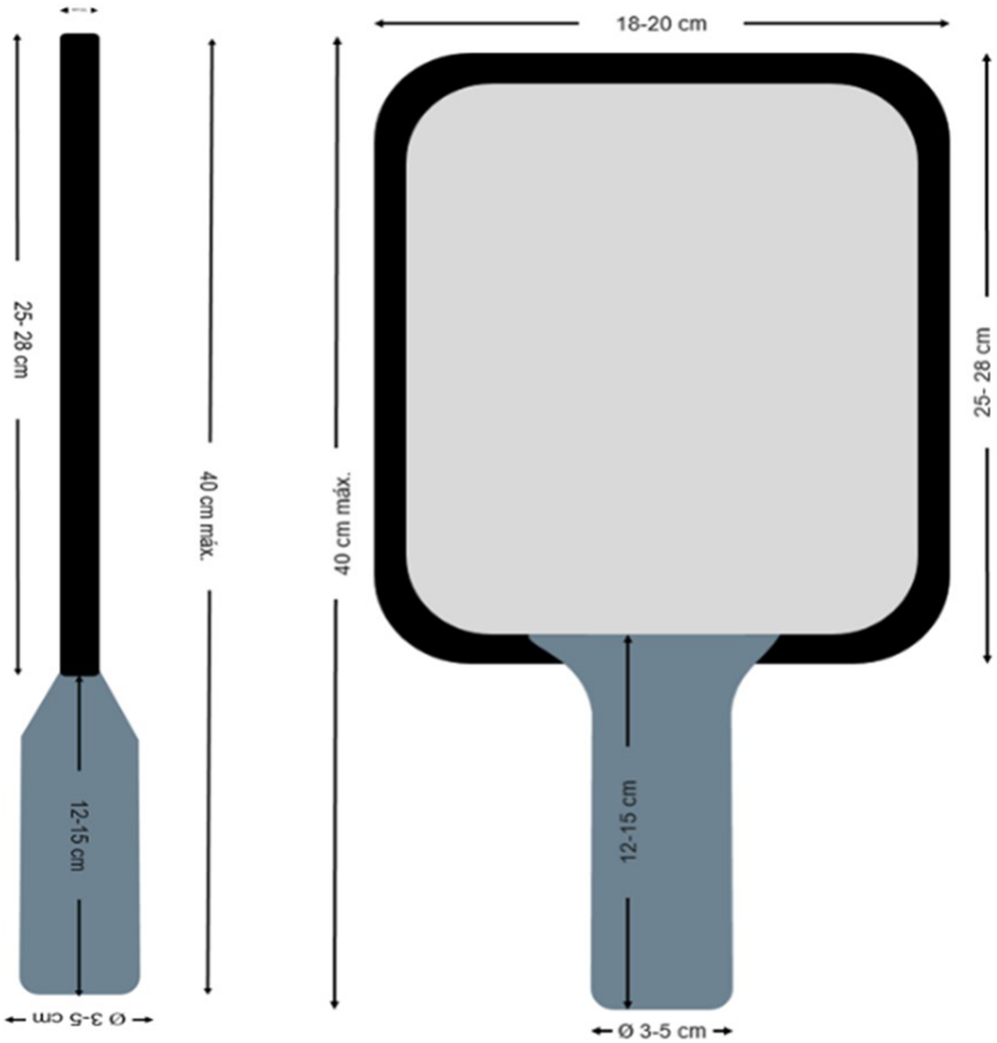
Cornerball paddle.

**Figure 5:**
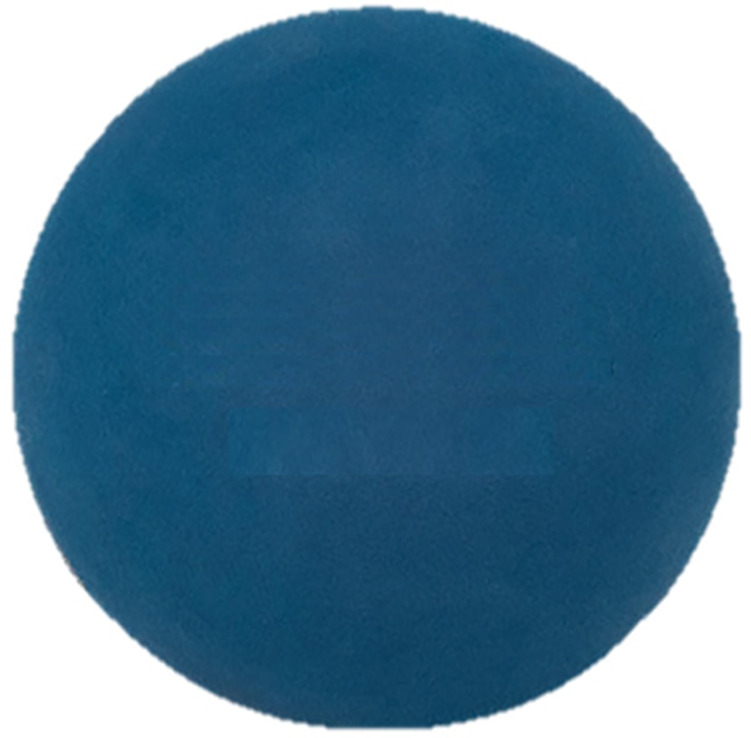
Cornerball ball.

(a) The serve is executed from behind the baseline, bouncing once inside the court.
(b) During the serve (see Figure 5), the ball must be at waist level or below, and the player must keep at least one foot in contact with the ground.
(c) After the serve, the ball needs to hit the wall on the serving player's side of the court. Once it touches that wall, it must bounce off the opposite wall and cross the “no bounce zone” area (see Figure 4) for the point to continue.
(d) While serving, the player cannot walk, run, or jump. Small foot movements that do not affect the initial position are allowed.
(e) The serve is considered made the moment the ball is hit or attempted to be hit, even if unsuccessful.
(f) The team that has the right to serve for the first point of each set decides which of its members will start the serve. The serving rights always belong to the team that lost the previous point, preventing consecutive winning serve points, as the winning team gives the serve to the opposing team. Also, the serve alternates between the members of the pair as long as the point is lost.
(g) The serving player must not serve until the receiving player is ready. However, the receiving player must adapt to a reasonable pace set by the server and be ready to receive the serve when the server is ready to execute it.

#### 3.4.3 Rule 3: service fault

(a) The serving player fails to hit the ball at all in their attempt.
(b) The ball bounces out of the reception area or within the “no bounce zone,” including the lines that define this area (the lines are considered in play).
(c) The ball hits the server, their partner, or any object they carry.
(d) If the ball, after hitting the double wall, touches the net before bouncing in the opposing court, it's considered a let, and the serve point is repeated.

#### 3.4.4 Rule 4: the return of service

(a) The receiving player must wait for the ball to bounce within their service reception area and hit it before it touches the ground a second time.
(b) The pair receiving the serve for the first point of each set will decide which of the two will start the return. Subsequently, there will be an alternating returner, mirroring the server's pattern. The last player to intervene in the winning team cannot return the serve. This avoids individual matchups during the set.
(c) If the ball hits the player receiving the serve or their partner, or if they touch it with the paddle before it hits the ground, a point is awarded to the serving team.

#### 3.4.5 Rule 5: ball in play

The ball is considered in play from the moment it is hit by the serving player until the referee calls let or decides the point, passing the serve to the corresponding team. Players must alternate hitting the ball.

#### 3.4.6 Rule 6: interference

Interference in the game occurs when a player intentionally or unintentionally impedes or obstructs the opponent's hit. In the former case, the point is awarded to the opponent, and in the latter, the point is replayed.

If the in-play ball hits an object not part of the game, such as another ball or any external element on the opponent's court, it is considered interference, and the point is replayed.

#### 3.4.7 Rule 7: lost point

A player or pair will lose a point if:

(a) A player, their paddle, or any object they carry touches any part of the net, including the posts, or the opposing court while the ball is in play.
(b) The ball bounces twice on the court before being returned by the corresponding player.
(c) The ball bounces out of the court boundaries, both vertically and horizontally.
(d) The ball is hit with a body part other than the paddle.
(e) The ball is hit more than once by a player before touching the wall (double hit).
(f) The net, paddle, clothing, or any body part touches it after hitting the ball.
(g) The ball bounces on their own court before touching the wall.
(h) The ball fails to touch both walls (own and opposing) before bouncing in the opposing court.
(i) The ball hits their wall, the opposing wall, and returns to their own court without bouncing first in the opposing court.
(j) During the serve, the player steps on the line or the court.
(k) During the serve, the ball doesn't bounce inside the court before being hit by the serving player.
(l) Only one player of the pair can hit the ball during the return. If both players simultaneously or consecutively hit the ball, they will lose the point.
(m) The player commits a fault on their first serve.
(n) The paddle is not held by the player at the time of the hit.

#### 3.4.8 Rule 8: won point. ULE 8

(a) If the ball, after bouncing in the opposing court, cannot be returned correctly by the opponent, the point is awarded to the player or pair that made the winning shot.
(b) If the hit ball touches both walls, bounces in the opposing court, and returns to the same court from where it was hit, the point goes to the player who made the hit.
(c) If the ball bounces in the opposing reception area and touches the net before the second bounce or the opponent's hit.

#### 3.4.9 Rule 9: double point

It's possible to score 2 points if the ball is hit before it touches the ground (volley), bouncing off both walls and achieving a point by hitting one's wall, the opposing wall, and then double bouncing in the opponent's court. In all other instances, the scored point will be 1.

### 3.5 Cornerball: behavior and discipline

Each competitor must maintain respectful and courteous behavior at all times while in the setting of any competition, even if not actively playing, showing consideration toward all individuals present. It is prohibited for players to forcefully throw the paddle or ball in any direction outside the court or execute an aggressive pass across the net when the ball is not in play.

In the same vein, both coaches and players must uphold appropriate behavior, understanding that penalties imposed by referees during the match will accumulate. Once the match commences, the game must proceed without interruptions, and no player can delay it without a valid reason that exceeds the established time limits.

Furthermore, aggressive behaviors, attitudes, and gestures contrary to the sportsmanship spirit from players, especially when directed at the Chief Umpire, referee, opponents, teammates, spectators, or anyone involved in the tournament, will be considered verbally abusive conduct. This encompasses insults and any oral expression implying evident disdain or mockery, even if not a direct insult.

Any violation of these mentioned aspects during the match will be penalized by the tournament's Chief Umpire according to the following classification:

• First offense: Warning (yellow card).
• Second offense: Warning with point loss (orange card).
• Third offense: Warning with disqualification (red card).

It's important to note that infractions committed by both members of the pair, and even their accredited coach, will be cumulative. Furthermore, the Competition Committee may impose additional penalties for the same incident as per the Sports Discipline Regulations.

In cases of extremely serious infractions, such as physical or severe verbal aggression, the Judge/Referee has the authority to immediately disqualify the player or coach responsible for the offense. If disqualification occurs during a match, the player will lose the match and must exit the competition. If the disqualification involves a coach, captain, or registered and accredited player in the tournament, they will be disqualified and must leave the competition.

### 3.6 Cornerball: methodoloy strategies

In order to implement the teaching of Cornerball in physical education classrooms, a series of methodological strategies are proposed for its instruction. One of the primary strategies to teach Cornerball involves utilizing small-sided games, mini-games, or task-oriented play, facilitating the acquisition of technical-tactical actions for all students, including those less skilled (67).

Additionally, increasing the height of the net can slow down the game, aiding in reaction time and enabling the recovery of optimal playing positions (68).

Furthermore, the Teaching Games for Understanding (TGfU) model by Bunker and Thorpe (69) could be employed, aiming to teach this new activity where students comprehend how to play and solve real game situations. This begins with modified and simple rules to ensure game continuity, adapting them to the psycho-physical characteristics of individuals (64). This approach allows students to assimilate and understand the nuances of the new activity. It helps foster tactical thinking and an awareness of game situations, as well as develop an appropriate language to express these situations (70).

Devís and Peiró (71) suggest that adaptable materials in racket sports play a crucial role. For instance, using a larger or lighter ball can reduce the speed of the game until students acquire sufficient skills to transition to standard equipment (68). Other elements that can be modified, aside from equipment, include playing without a net, using paddle rackets, allowing for double bounces, among others.

Moreover, another available methodological strategy for teaching and motivating students in this proposed activity is the Sport Education model proposed by Siedentop (72, 73). This model organizes the structure akin to a sports season. Students are organized into teams, fostering a sense of belonging to a group, instilling values of group cohesion, and acquiring social competencies and skills. Additionally, it involves a temporal organization with a competition schedule and a record of actions performed by the students.

## 4 Conclusions

Physical Education, much like the educational system, must progress, change, and adapt to the shifts and advancements occurring in today's society. Employing new methodologies, resources, or innovative teaching approaches to motivate students and capture their attention during classes is crucial. This ensures they can undergo an adequate teaching-learning process and, consequently, acquire the necessary knowledge to navigate contemporary society. It is from this need that Alternative Activities (AAs) emerge as a key content to teach in Physical Education (PE), fostering the holistic development of individuals.

Thus, the alternative sport Cornerball is presented as a game directly applicable within physical education classes. This sport enables the exploration of diverse motor situations and various social contexts.

Cornerball not only contributes to physical and motor benefits but also enhances fundamental physical capacities. It develops perceptual-motor abilities by addressing spatial-temporal control and bodily coordination, foundational elements supporting overall coordination and equilibrium.

Ultimately, there's an expressed need for further research and interventions in Physical Education classes to demonstrate the utility of these alternative sports. This aims to underscore the necessity of integrating them into the teaching-learning process to facilitate the acquisition of new knowledge and skills.

## Data Availability

The original contributions presented in the study are included in the article/Supplementary Material, further inquiries can be directed to the corresponding author.
